# New York Academy of Medicine

**Published:** 1889-05

**Authors:** 


### NEW YORK ACADEMY OF MEDICINE.

Section in Laryngology and Rhinology.

*Meeting of March 26th, 1889.*

### COMPLETE BONY OCCLUSION OF THE ANTERIOR NARES DUE TO HYPEROSTOSIS OF THE FACIAL BONES.

Dr. G. B. Hope presented a case of this rare affection, occurring in a young girl, with an account of the operative procedures that were being carried out to secure a nasal passage. The occlusion of the nares was due to growth of the turbinated bones, but while other bones of the face had attained corresponding size and weight (the lower jaw being three-fourths of an inch in thickness and the roof of the mouth half an inch), the other passages and foramina of the skull did not seem to have been encroached upon, with the exception of the lachrymal canal, which had become obstructed first of all, and the Eustachian tube, which was enlarged and of bony hardness. The smell, sight, and hearing were good. She had some cough from pharyngeal catarrh, snored at night, breathed almost entirely through the mouth, and had frequent attacks of coryza. Her other functions were normal. Of nine children in her family, she was one of two living. Her breathing had always been difficult, and she had had two attacks of pneumonia and one of bronchitis. With the electric trephine Dr. Rice was making an opening, now nearly completed, through to the pharynx.

Dr. Knight had seen thickenings of the palatal bones which about closed the posterior nares, but no case like the one presented. The slight impairment of nervous and other functions was remarkable considering the amount of bony growth.

### ETHMOIDITIS—ATTEMPTED OPERATION.

Dr. Knight presented a patient upon whom he had operated on the right side, without success, to remove a soft red mass growing in the upper back part of the nasal fossæ. The affection had begun a year or more previously, being ushered in by intermittent and then persistent headache, and being accompanied by loss of smell and taste. Acids had been without result; the cautery had made a passage, but it had soon closed up again; Weir's forceps had next been used, and had caused repeated discharges of pus, which applications of peroxide of hydrogen had not arrested as yet.

LARYNGEAL VERTIGO.

Dr. S. T. Armstrong, of the Marine-Hospital Service, read a paper with this title, in which he had collected all the recorded cases of laryngeal vertigo (sometimes called "epilepsy" and "syncope"), nineteen in number. He gave an elaborate review of the various theories which had been advanced to explain the occurrence of unconsciousness produced by laryngeal irritation. Cases had first been reported by Charcot. Dr. Lefferts and Dr. Gray had each published cases. Convulsions had in most instances been absent; in a few of the cases cited there had been movements. On emerging, the patient was not confused, and in one case it had been recorded that he would continue saying what the attack had interrupted him in. Local treatment of the larynx cured the whole trouble. These facts had led some to object to the term "laryngeal epilepsy," as not being properly descriptive. However, if we recognized that epilepsy might result from a scar or some persistent cause of irritation in any part of the body, it might not seem improbable that intense irritation of the superior laryngeal upon sudden sharp coughing might result in epileptoid unconsciousness by a direct effect on the brain. We need not suppose with some that the vagus and heart were affected primarily. Bromides had in some instances stopped the seizures. In a case occurring in the speaker's practice the attacks had been much favored by a condition of nervous exhaustion or the breathing of a vitiated atmosphere. Dimness of vision and muscæ volitantes preceded the attacks. The speaker preferred the term laryngeal syncope, as standing for a symptom of a functional rather than organic disorder.

THE CENTRAL TRACTS OF OLFACTORY NERVES AND THEIR DISEASES.

Dr. C. L. Dana read a paper with this title, in which he presented some facts and views concerning the first cranial nerve that, as he believed, were new. The nose was treated by rhinologists mostly as a respiratory organ. Disturbances of the sense of smell were commonly disregarded by physicians and patients, but it should be remembered that this sense served useful purposes. It was our most delicate means of appreciating attenuated matter. In fishes something akin to a sense of smell, if not an identical sense, was well developed. In reptiles there was a fair development of the sense, and in birds scarcely any; but the highest development was seen in mammals, except those of aquatic habits, like the seal, in which it was nearly wanting. In the anthropoid apes it was not well developed, and in savages, the negro and Indian (contrary to the assertions of some writers), its development was not equal to that which it attained in civilized races. The Indian might be superior in acuteness of smell, but in the discrimination of different odors and the appreciation of flavors and their æsthetic associations the civilized man was as much the superior of the Indian as the latter was in advance of the anthropoid ape. In animals it served for sexual purposes, for selecting food, for hunting prey, and for avoiding enemies. In man it served for selecting food and drink, for arousing the sense of hunger, for warning him against injurious vapors, and for æsthetic and to some extent sexual purposes. Diminution in keenness of olfaction was found present in the criminal, lunatic, idiotic, and degenerate classes in society. The olfactory sense in man was growing, and fortunately so, for every avenue which carried new impressions to the brain helped to develop it. He believed that the sense of smell and taste would one day be cultivated in children as a means of causing them to grow up with greater keenness of mind and larger capacity for discrimination and for æsthetic enjoyment. The sense of smell had always been appealed to in religious rites, and women instinctively learned that to exhale a delicate perfume added to their sexual attractiveness. He had lately observed anosmia in a number of neurasthenic patients, which was often independent of a catarrhal state, and he believed that, like asthenopia, it occurred in both neurasthenia and hysteria. In testing for smell he used ten phials, containing from one one-hundred-and-sixtieth to six-tenths of a grain of vanilline.

At this point the paper enters into an elaborate discussion of the anatomy of the olfactory nerve. In closing the paper the speaker said that he had constructed a qualitative olfactometer for testing the sense of smell much as we tested color-sense or appreciation of musical pitch, consisting of two sets of phials. In the first set were the monatomic alcohols of gradually increasing molecular weight (methyl, ethyl, propyl, and amyl alcohols), and also increasingly pronounced odor; and in the second set were the fatty acids (formic, acetic, propionic, butyric, and valeric acids), whose odor varied from a purely acetic smell to one in which flavor alone was present. A person with a normal sense of smell should be able to distinguish each of those odors and their relative place in the series. Some might be found having a keen sense of smell but odor-blind, just as we found people with keen vision who were color-blind.

Diseases of the intracranial olfactory tracts occurred in locomotor ataxia, general paresis, senile decay, and (of a functional sort) in hysteria. Olfactory neuritis was rare. The tracts might sustain injury in various ways, as from tumors, thrombi, hæmorrhages, and meningitis in the adjacent pia. Syphilis was probably the leading cause of anosmia.

He believed tests of the sense of smell might aid in the diagnosis of central organic diseases of the brain after the rhinologist had determined that there was no nasal condition affecting the peripheral end organs of the olfactory nerve.

The Chairman, who with others discussed the nasal causes of loss of smell, said that, where it was due to hypertrophied conditions in the parts or to polypi, recovery might be brought about, but not after the atrophic stage had been reached.

---

### THE OBSTETRICAL SOCIETY OF BOSTON.

*Meeting, February 9, 1889.*

Dr. J. S. Greene, of Dorchester, read a paper entitled

### SOME USES OF THE VAGINAL TAMPON IN OBSTETRIC ART.

The following is a brief abstract of the paper:

The soothing and moderating influence of the vaginal tampon in dysmenorrhœa and menorrhagia, and the relief which it gives in reflex nervous disorders in cases of pelvic irritation, has suggested its use in cases in obstetric practice, and the report of the following cases is offered in the hope of eliciting accounts of similar cases.

The tampon as I have applied it for purposes here cited has generally consisted of the flattened disks of absorbent cotton moistened with glycerine such as are used in gynæcology. The patient lies on her left side and these pieces, held in the grasp of long, slender forceps, are successively introduced by the right hand, and are guided to their place and adjustment by one or two fingers of the left hand in the vagina. When placed, these pieces form a layer upon the posterior vaginal wall from the cul-de-sac to the verge of the outlet, where they are sustained and secured by a larger piece on either side of the urethra behind the os pubis. No strong pressure is applied in packing, the guiding finger is preferred to the speculum, no undue distention of the vagina is produced, and the packing is no firmer than is thought to be needful to prevent it from being easily forced out.

The degree of firmness required for this purpose is sufficient to impart considerable support to the uterus and at the same time is comfortable to the wearer. The tampon as thus used has no correspondence except in name with that packing of the vagina in use to control hæmorrhage during abortion. With or without good reason the latter proceeding has the reputation of promoting the evacuation of the uterus by stimulating uterine contraction; but it does this, if at all, by imprisoning the flow, distending the vagina, and pressing downward the perinæum.

On the other hand the effect of the tampon as I now advocate it is to increase the secretions, while not hindering their exit, to allay irritation, relieve engorgement, and impart just that degree of support without distention which promotes, rather than opposes, comfort.

When hæmorrhage appears in the course of pregnancy I find the tampon, thus applied, a valuable resource for arresting instead of advancing a threatened abortion.

Rest in bed, aided perhaps by an opiate, or perhaps by hot douching, will often suffice to avert the mischief: but what if the patient does not see the necessity of these precautions, and insists on going about?

A young woman half-through the period of her first pregnancy came to my office, having had flow enough to continue occasional change of napkins for a fortnight. The cervix uteri was flaccid and widely patulous up to the internal os, and the cervix and vagina were catarrhal. She also had reflex symptoms, especially vomiting. I painted the cervix inside and out and the roof of the vagina with a strong solution of iodine, and put in a vaginal tampon firm enough to contribute some support to the uterus. A few days later these proceedings were repeated at my office. Entire relief of all the symptoms, both local and reflex, followed this treatment: and the patient kept safe and sound to the end of gestation.

During the night of August 30, 1887, I was called to care for a young woman with a sharp attack of secondary hæmorrhage after confinement. She had been comfortably delivered of her third child eleven days previously. She was tall, rather spare, inclined to anæmia, and had been overworked. I could recognize no exciting cause. Delivery and subsequent care had been conducted antiseptically, and I was confident that the placenta and membranes had been wholly expelled. This attack ceased, and if any lochial discharge continued it was without odor.

She was given ergot and quinine and douched with hot water. A week later another hæmorrhage occurred. I then resorted to the vaginal tampon, renewing it twice in the ten days next following. The patient had no further trouble.

I cannot assert that in either of these cases the same favorable course would not have been exhibited if no tampon had been used. I only mention them as experiences which strengthened in my own mind the good opinion I had formed *a priori* of the tampon as an available resource in obstetric practice, and as illustrations of certain conditions wherein its use seemed suggested by analogies derived from gynæcological procedure.

For many years I have highly valued the hot vaginal douche in cases of this kind. I have never found any good from medicines, and I do not believe in stretching the cervix uteri.

From recent experiences I have come to the belief that the vaginal tampon as an alternative or substitute for the douche, is an invaluable resource. Its combined depleting and supporting influence at once removes reflex irritability, enabling the stomach to do its full duty, and permits walking and other forms of exercise with the minimum of fatigue, thus securing the two great essentials of good nutrition.

The last case which I shall present as illustrative of the obstetric uses of the tampon is a typical instance of the class of patients above denoted.

Mrs. Y. is of light blonde complexion, twenty-two years of age. Six years ago she relinquished systematic study for want of strength, and was for some months under my guidance for neurasthenia. She improved but continued easily susceptible of fatigue; married in October, 1886.

Went to Europe and returned in October, 1887, with symptoms of pregnancy.

Nausea and vomiting became troublesome, headaches followed, and the shadow of impending prostration darkened the background of the prospect. Vaginal douches were used and one or two medicines tried before October ended; but no advantage was gained. The vaginal tampon was resorted to November 1st, in the eighth week of pregnancy, and its good effect was immediately manifest.

She resumed her daily walks, retained her food, improved in appetite, and slept better. The tampon was renewed at intervals of five to seven days for nearly five weeks. When discontinued it was with the purpose of resuming its use if any retrograde tendency should be noted; but the advantage gained proved a lasting one. She had entered upon the fourth month of her pregnancy, and taught by much experience of weakness to be prudent and to carefully follow a prescribed policy, she passed safely, and on the whole comfortably, through the subsequent months of gestation.

Dr. Abbott asked whether the tampon was used empirically or for the purpose of supporting the uterus.

The reader replied that it acted partly as a depleting agent, relieving engorgement, and partly mechanically as a support.

Dr. Davenport said that he was very much interested in the paper, as he had very recently been engaged in enumerating and formulating the various uses of the tampon in gynæcological practice. These obstetrical uses of the same agent had not occurred to him at the time, but they completed the consideration of the subject. Most of the uses spoken of were rational, and might be used with great benefit in the conditions named. Of especial value was the use of the tampon in the early months of pregnancy, to allay vomiting, and relieve pressure. Even where the vomiting was not excessive but only annoying, he thought a light tampon might afford great relief. The depleting effect of the glycerine would not in his opinion be sufficient to do any harm by interfering with the circulation of the uterus.

He had recently had a case illustrating the value of this method following miscarriage. The patient had miscarried on January 10, and had kept quiet for ten days without any unpleasant symptoms. She resumed her business, which was that of a fashionable dressmaker, and in a few days came complaining of backache and slight hæmorrhage. Dr. Davenport found a heavy retroverted uterus, with a badly lacerated cervix, and with a small amount of blood and mucus exuding from it. Twice packing with the glycerine tampon checked the flowing, restored the uterus to its normal position, and relieved the backache. Then a pessary was applied which will be worn until the cervix is repaired, which will be in the course of a few months.

In reply to a question by Dr. Richardson, the reader said that he had used the tampon in the case reported of threatened miscarriage at the fourth month, not as a plug to stop hæmorrhage, but to give support to the uterus, and relieve the existing inflammation of the mucous membrane, and that its use was to a certain extent empirical.

Dr. Richardson had never used it in these cases, and would think that the packing might start up pains, or, by causing a backing up of blood, there might be danger of separation of the placental tissue from the uterus. He had used the tampon during pregnancy only for the purpose of relieving nausea and vomiting. He had recently been interested in some German accounts, where the uterus itself was packed with iodoform gauze.

Dr. Green said he could well believe that a light, medicated tampon might sometimes be of service in various affections of pregnancy, either for obtaining the derivative effect of glycerine, or for healing erosions or diminishing undue sensitiveness of the cervix. But applied to the extent of affording appreciable mechanical support, he believed the tampon was an unsafe expedient. He had himself, when a hospital house-officer, induced an abortion in a patient not supposed to be pregnant, by applying a tampon to raise a retroverted uterus. And he believed one of the most effective means of exciting uterine contractions was distending the vagina with tampon or colpeurynter. He inferred, therefore, that the reader could not have employed the usual gynæcological tampon; and that his good results were attributed to local medication, and not to mechanical support, which implies a certain amount of vaginal distention.

The reader agreed with Dr. Green, that in the case of distention of the vagina by the tampon there would be risk of inducing labor, but said he had no hesitation in using the tampon in the manner described by him in any parturient case.

Dr. Cotting then read a paper entitled

THE LIE OF THE FŒTUS.

Dr. Alfred Worcester, of Waltham, read by invitation a paper entitled

A SERIES OF TWO HUNDRED CONSECUTIVE CASES OF MIDWIFERY IN PRIVATE PRACTICE.

Dr. Homans showed a fibroid tumor of the uterus, weighing eight pounds, removed by hysterectomy, with recovery. Also the fallopian tubes from a case of salpingitis removed entire. The tubes were each about seven inches long, and one and three-fourths inches broad, distended with pus.

Dr. Sinclair reported

A CASE OF PARTIAL RETENTION OF THE MENSTRUAL EXCRETION BY A MEMBRANOUS OCCLUSION OF THE OS UTERI.

On the 13th of October last a lady consulted me about menstrual disorder. From the 25th of December, 1887, the date of her delivery, until about the same date in September, 1888, a period of nine months, her menses failed to appear, although she had sensation of being unwell from time to time, which gradually died away without any visible discharge. At this time she had a gush of blood from the vagina which lasted two, or maybe three hours. At the time of her visit on October 13, 1888, there was a slight, brownish, mucous discharge from the vagina. Her general condition seemed good. Appetite, bowels, sleep, and kidneys well. No complaint made of pelvic organs. On tactile examination, I found an enlarged and boggy cervix uteri, and a heavy uterine body. The examining finger was stained with a light brownish substance. The question of pregnancy naturally occurred to me. Specular examination revealed an unusual state of things—an orifice of the size of a cambric needle from which exuded a stringy substance, which I at first mistook for the product of an abscess. Into this small opening I passed a small probe, at the same time pressed the speculum against the cervix uteri, with the result that more of the substance referred to was squeezed out. On further advance of the probe, I found that it entered freely into a cavity of unknown depth. I then made free incision with a bistoury, followed by a gush of four or even more ounces of a grayish, stringy fluid, mucus, and blood. I simply incised the occluded os uteri. The uterus measured over four inches in depth. The uterine cavity was washed out and the parts thoroughly cleansed by a solution of corrosive sublimate.

The after treatment of such a case was obvious, and may be summed up in the prevention of recurrence. It would seem to one not acquainted with the behavior of such cases that little else than that I had done was necessary. My experience is that of others, that the tendency to recurrence of occlusion of the os uteri in such cases is constant and demands care. The treatment consisted in dilatation with the uterine dilator, and by iodized phenol to the endocervix. The uterus is now three inches in depth, and the catamenia have become regular. The patient may need watching. Cases of this kind are not common, and in thirty years only four have come to my care.

